# Influence and Correction from the Human Body on the Measurement of a Power-Frequency Electric Field Sensor

**DOI:** 10.3390/s16060859

**Published:** 2016-06-10

**Authors:** Dongping Xiao, Huaitong Liu, Qiang Zhou, Yutong Xie, Qichao Ma

**Affiliations:** State Key Laboratory of Power Transmission Equipment & System Security and New Technology, Chongqing University, Chongqing 400044, China; 20141102032@cqu.edu.cn (H.L.); 20131101031@cqu.edu.cn (Q.Z.); 20151102059t@cqu.edu.cn (Y.X.); 20151113002t@cqu.edu.cn (Q.M.)

**Keywords:** power-frequency electric field, portable measurement, human body influence, correction coefficient, linear fitting

## Abstract

According to the operating specifications of existing electric field measuring instruments, measuring technicians must be located far from the instruments to eliminate the influence of the human body occupancy on a spatial electric field. Nevertheless, in order to develop a portable safety protection instrument with an effective electric field warning function for working staff in a high-voltage environment, it is necessary to study the influence of an approaching human body on the measurement of an electric field and to correct the measurement results. A single-shaft electric field measuring instrument called the Type LP-2000, which was developed by our research team, is used as the research object in this study. First, we explain the principle of electric field measurement and describe the capacitance effect produced by the human body. Through a theoretical analysis, we show that the measured electric field value decreases as a human body approaches. Their relationship is linearly proportional. Then, the ratio is identified as a correction coefficient to correct for the influence of human body proximity. The conclusion drawn from the theoretical analysis is proved via simulation. The correction coefficient *k_b_ =* 1.8010 is obtained on the basis of the linear fitting of simulated data. Finally, a physical experiment is performed. When no human is present, we compare the results from the Type LP-2000 measured with Narda EFA-300 and the simulated value to verify the accuracy of the Type LP-2000. For the case of an approaching human body, the correction coefficient *k*_b_* = 1.9094 is obtained by comparing the data measured with the Type LP-2000 to the simulated value. The correction coefficient obtained from the experiment (*i.e*., *k*_b_*) is highly consistent with that obtained from the simulation (*i.e*., *k*_b_). Two experimental programs are set; under these programs, the excitation voltages and distance measuring points are regulated to produce different electric field intensities. Using *k*_b_ = 1.9094, the corrected measurement of electric field intensity can accurately reflect the original environmental electric field intensity, and the maximal error is less than 6% in all the data comparisons. These results verify the effectiveness of our proposed method.

## 1. Introduction

Strong electric fields exist around high-voltage electrical equipment. Existing research shows that strong power-frequency electric fields have potential harmful effects on human health and safety [1]. Therefore, relevant institutions and international organizations have developed standards or have recommended limitations regarding power-frequency electric fields for the general public and power system practitioners, as shown in Table 1 [2,3,4,5,6]. Here, the spatial electric field in the absence of any object is considered.

However, power system practitioners are likely to be exposed to strong electric fields in a short amount of time because of their handling of high-voltage electrical equipment. Excessively strong electric field intensities result in discomfort and panic for practitioners, and such effects are harmful to personal safety and can lead to disoperation [7,8,9]. If practitioners can be equipped with a portable safety protection instrument that can measure the electric field intensity of a working area in real time and issue a safety precaution, then the safety and health of practitioners in the workplace can be ensured. Our group has been working toward this objective.

Several power-frequency electric field measuring instruments are available for commercial purposes [10]; they include the Narda EF series of Germany, the PMM of Italy, and the CA of France. In addition, many research teams have studied and developed electric field measuring instruments for special purposes [11,12,13]. Most current electric field measuring instruments require bracing the probe with an insulated bracket; they also require measuring technicians to be far away from the probe to avoid the influence of human body proximity on measurements [14]. However, the electric field warning devices developed by our team need to be carried by working practitioners, who thus become inevitably involved and exert a potential effect on measurements. Electric field intensities measured in real time are used as the main index of safety precaution. Meanwhile, the original environmental electric field intensity subjected to exposure limits and the influence of the human body during measurement are interesting topics that are worth exploring.

The influence of the human body on electric field measurement has attracted increasing attention in recent decades. In [15], the existence of a human body was proposed to make the spatial electric field change in a certain range. In [16], the changes in the electric field distribution on the different parts of the human body when the body was isolated from the ground were demonstrated. In [17], the proportional relation between undistorted electric field and distorted electric field at certain points on the surface of a human body model was presented. However, these studies only showed that human body occupancy leads to the distortion of spatial electric fields; they did not focus on the influence of human body proximity on electric field measurement and the ways to correct measured results.

In the present work, we build an equivalent circuit that explains the principle of electric field measurement on the basis of a single-shaft electric field measuring instrument called a Type LP-2000 [8], which was independently developed by our research team. Then, simulation via Ansoft/Maxwell is conducted. The influence of the human body on the electric field measuring sensor of a plate capacitor is discussed theoretically, followed by a proposal of a correction coefficient for the influence of the human body on electric field measurements. In the laboratory, the electric field generated by different excitation voltages in the absence of a human body is measured with a Type LP-2000 and Narda EFA-300. The numerical comparison and error analysis of the measured data and simulated data verify the correct measurement of the Type LP-2000. Thereafter, electric fields are measured with a portable Type LP-2000 that is fixed on an individual’s arm under two sets of experimental programs. The corrected electric field values obtained by using the real-time measured values multiplied by the correction coefficient are compared with the original electric field in the simulation environment; the errors are subsequently analyzed.

## 2. Equivalent Circuit Analysis of the Influence of the Human Body on Electric Field Measurement

### 2.1. In the Absence of a Human Body

The core of Type LP-2000 comprises a couple of metal polar plates that can be seen as a capacitor. Inductive charges with the same frequency appear on the metal plates when the measuring sensor is put into the alternating electric field. Then, the induced voltage can be measured. The relation between the induced voltage and the electric field intensity at the measuring point is linearly proportional when the physical dimension of the planar plate capacitor is sufficiently small [18].

(1) $${\dot{U}}_{C}=D\dot{E}$$

where *D* is the distance between two polar plates and ${\dot{U}}_{C}$ and $\dot{E}$ are the induced voltage and electric field intensity in phasor form respectively.

**Figure 1:**
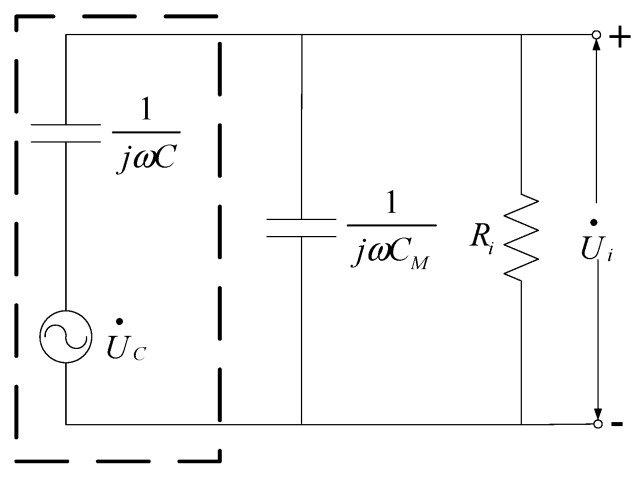
Equivalent circuit of the measuring system.

The equivalent circuit of the measuring system for obtaining the induced voltage is shown in Figure 1.

In Figure 1, the induced voltage is equivalent to a voltage source $\overset{•}{U_{C}}$ with angular frequency ω, *C* is the inherent capacitance of the plate capacitor, *C_M_* is the capacitance of the measuring capacitor, *R*_i_ is the input resistance of the measuring circuit, and $\overset{•}{U_{i}}$ is the output voltage. Therefore,

(2) $${\overset{•}{U}}_{C}=\frac{1+j\omega R_{i}(C_{M}+C)}{j\omega R_{i}C}{\overset{•}{U}}_{i}$$

By further processing the circuit, the RMS value *U_irms_* of $\overset{•}{U_{i}}$ can be obtained. The RMS value *U_crms_* of $\overset{•}{U_{C}}$ is:

(3) UCrms=1+ω2Ri2(C+C)M2ωRiCUirms

By combined Equations (1) and (3), we obtain the RMS value *E*_rms_ of the electric field, that is,

(4) Erms=1+ω2Ri2(C+C)M2DωRiCUirms

In the measurement circuit, the measuring capacitance is at the nF-level and is much larger than the inherent capacitance of the sensor, *i.e.*, *C_M_* >> *C*; thus, Equation (4) can be simplified to

(5) Erms=1+ω2Ri2C2MDωRiCUirms=KUirms

where *K* is a ratio coefficient written as

(6) K=1+ω2Ri2C2MDωRiC

In Equation (5), ω, *R_i_, C, C_M_*, and *D* are all fixed values; thus, K is a constant. When the parameters of the measuring circuit are all fixed, the electric field intensity in the location of the parallel-plate capacitive sensor is proportional to the output voltage of the sensor. The electric field intensity *E*_rms_ of the observation point can be obtained by measuring *U*_irms_.

### 2.2. In the Ca**s**e of an Approaching Human Body

In practical measurements, when an individual approaches the sensors, the measured electric field intensity changes because the human body possesses complex tissues. The relative dielectric constant of living tissue in a power—frequency environment is about 10^5^–10^6^, and the conductivity is about 0.1 S/m [19]. The human body can be regarded as a conductor; therefore, charges gather on its surface as it remains in the electric field, and such accumulation affects the spatial distribution of the original electric field [20].

**Figure 2:**
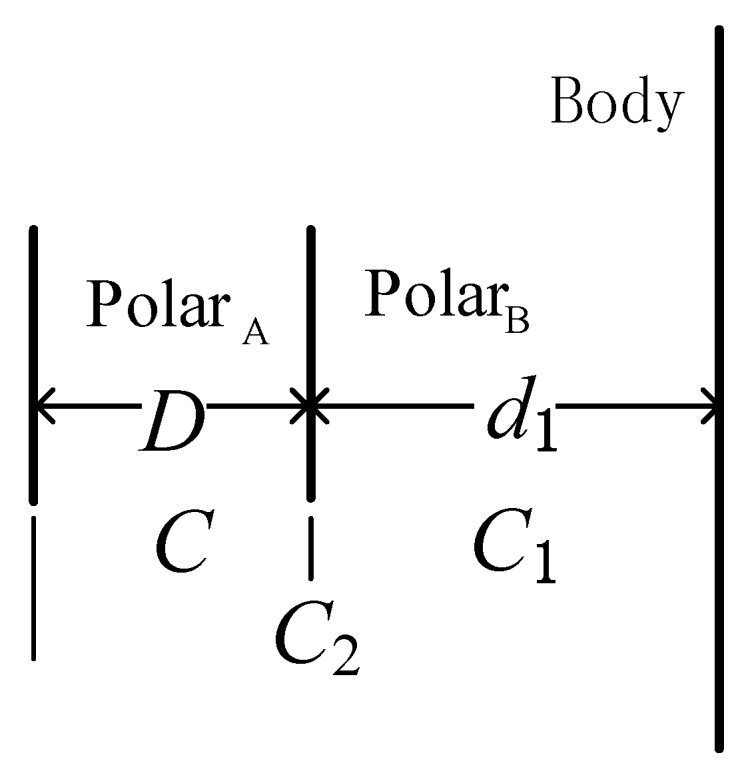
Relationship between the human body and the polar plates of the capacitor.

The relationship between the human body and the measuring sensor of the plate capacitor is shown in Figure 2.

**Figure 3:**
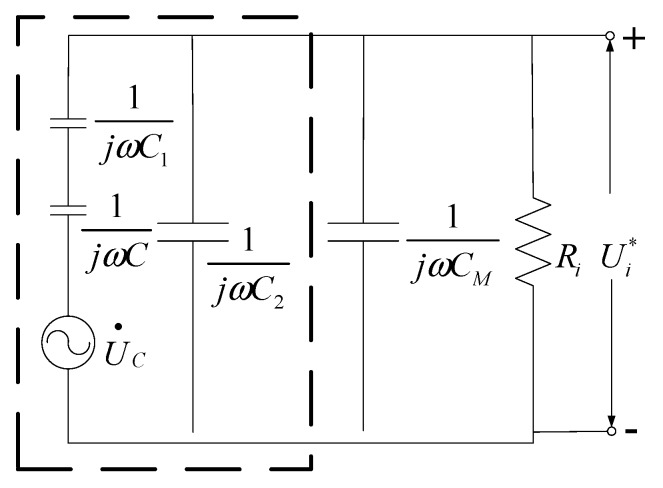
Equivalent circuit of the measuring system in the case of an approaching human body.

In Figure 2, Polar_A_ and Polar_B_ are the two polar plates of the capacitor, and *D* is the distance between the two plates, with the gap filled with epoxy resin. The distance between the human body and Polar_B_ is d_1_, with an air medium in the middle. The body with Polar_A_ and body with Polar_B_ form the equivalent capacitors, *C*_1_ and *C*_2_, respectively. The equivalent circuit of the measuring system in the case of an approaching human body is shown in Figure 3.

According to Figure 3, *C_M_* is much larger than *C* and *C*_1_; therefore,

(7) Erms=1+ω2Ri2(C+MC2)2DωRi(C1CC1+C)Ui*rms=KbUi*rms

where *K_b_* is also a ratio coefficient written as

(8) Kb=1+ω2Ri2(C+MC2)2DωRi(C1CC1+C)

Equation (7) shows that when the measuring parameters are fixed, *K_b_* is a constant, and the output voltage of the sensor remains proportional to the electric field at the measuring point.

By comparing Equations (5) and (7), we observe that *C_M_* + *C*_2_ > *C_M_* and (*C*_1_*C*)/(*C*_1_ + *C*) < *C*_1_, *K_b_* > *K*. Moreover, we note that U_i_*_rms_ < *U*_irms_ if *E*_rms_ is constant. In other words, the measured induced voltage decreases when a human body approaches the electric field measuring sensor. Without correction, the measured electric field intensity becomes smaller than the physical truth.

In sum, the correction coefficient *k_b_* can be defined as

(9) $$k_{b}=\frac{K_{b}}{K}$$

Thus, the spatial electric field intensity in the absence of a human body can be obtained by using the electric field intensity measured with the portable Type LP-2000 in real time multiplied by the correction coefficient *k_b_*.

## 3. Simulation Analysis

### 3.1. Simulation Model Setting

The simulation model is set up with the software Ansoft/Maxwell, and most of the simulation parameters are set on the basis of the physical experiment condition shown in Section 4.

Different electric field intensities are produced when different levels of sinusoidal voltage with power-frequency are applied to the power transmission line. In the simulation model, the power transmission line is a copper conductor with a diameter of 14 mm and a distance of 122 cm from the ground. Observation point P is at the same level as the conductor and at 53 cm from the conductor in the horizontal distance.

**Figure 4:**
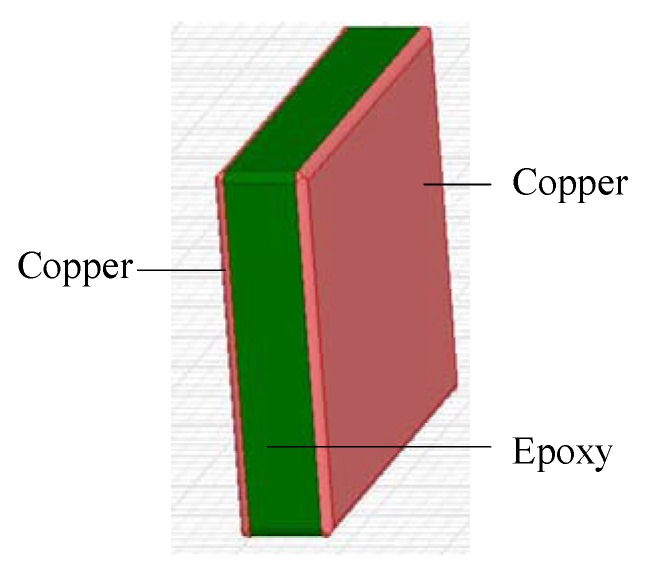
Simulation model of measuring sensor.

The simulation model of the plate capacitor is shown in Figure 4. The two polar plates are made of copper, and measure 50 mm in length, 36 mm in width, and 1 mm in distance. The filling medium is epoxy resin. The center of the sensor is coincident with the observation point P.

In addition, the condition of the bottom boundary is set as grounding, and the boundary conditions of the other five surfaces are set as the balloon boundary with the electrical potential set to zero at infinity during the simulating.

### 3.2. Simulation Results and Analysis in the Absence of a Human Body

**Figure 5:**
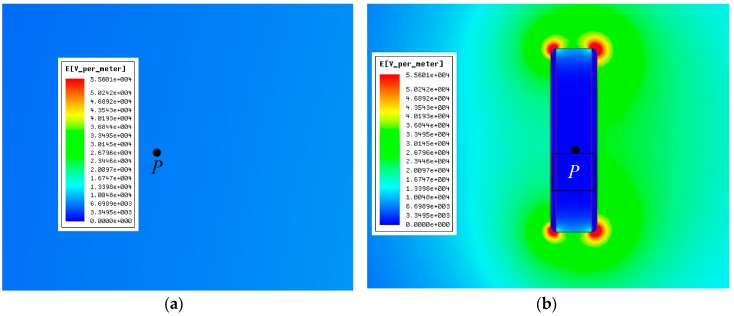
Electric field spatial distribution surrounding observation point *P* (no influence of a human body). (**a**) Before placing the sensor; (**b**) after placing the sensor.

Figure 5 shows the simulated results of the electric field spatial distribution surrounding the observation point P before and after placing the measuring sensor under the same excitation voltage conditions.

Figure 5 shows that the original electric field surrounding the observation point *P* is evenly distributed. After the placement of the sensor, the electric field at the four corners of the sensor become significantly distorted and enhanced, whereas the surface electric field outside of the polar plates is slightly enhanced [21,22]. The electric field at observation point P is still even, whereas the electric field intensity decreases relative to the original value because of the shielding effect of the polar plates.

Table 2 shows the simulated results of the original electric field *E*_0_ and electric field *E*_rms_ after placing the measuring sensor at point P when different levers of excitation voltage are generated.

**Figure 6:**
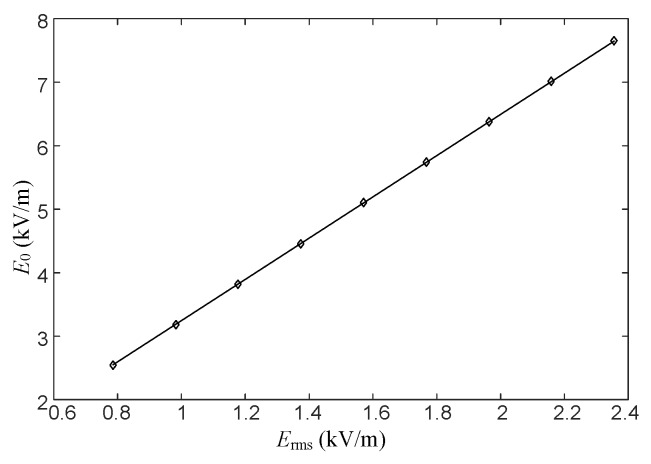
Relation curve of the electric field values at point *P* before and after placing the measuring sensor.

The data in Table 2 are linearly fitted, as shown in Figure 6.

Figure 6 shows that a linear relation exists between *E*_rms_ and *E*_0_. Their relation can be expressed mathematically as

(10) $$E_{0}=k_{0}E_{\text{rms}}$$

where k_0_ is the correction coefficient between *E*_0_ and *E*_rms_. Here, *k*_0_ = 3.2464.

### 3.3. Simulation Result and Analysis in the Case of an Approaching Human Body

We assume that the height of the approaching individual is 176 cm. Given that the individual’s shoes are isolated, the thickness of the insulating materials is assumed to be 2 mm during the simulation so that the person does not directly come into contact with the ground. The measuring sensor is fixed on the person’s arm, and its center is located at point P, similar to that shown in Figure 5.

**Figure 7:**
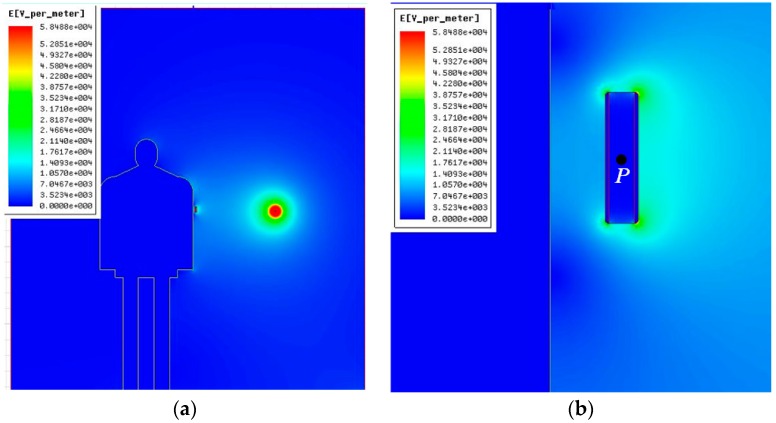
Electric field distribution in the case of an approaching human body. (**a**) Surrounding human body and conductor; (**b**) surrounding measuring sensor.

Figure 7a shows the electric field distribution surrounding the human body and conductor. Figure 7b shows the partially enlarged detail surrounding the measuring sensor.

As shown in Figure 7, the electric field spatial distribution outside the sensor is distorted further in the case of an approaching human body, whereas the electric field between the polar pates is evenly distributed.

Table 3 shows the simulation results of the electric field before and after the approach of a human body (*i.e*., *E*_rms_ and E*_rms_ respectively) at point P when different levels of excitation voltage are generated.

**Figure 8:**
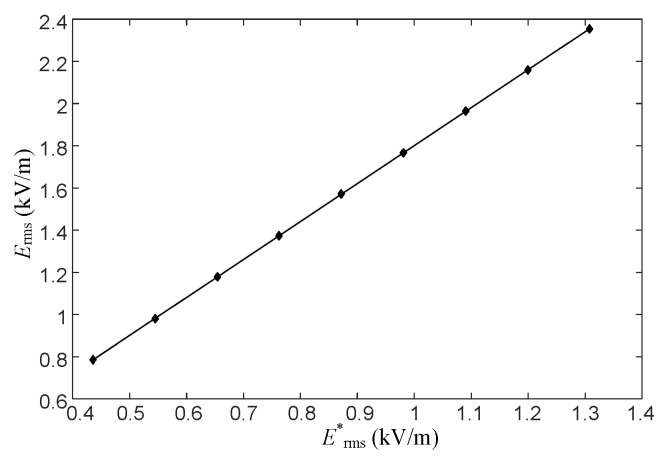
Relation curve of the electric field intensity at *P* before and after the approach of a human body.

The data in Table 3 are linearly fitted, as shown in Figure 8.

Figure 8 shows that after the approach of the human body, the electric field intensity at the observation point decreases but is proportional to that before the approach of the human body. This situation is explained in the aspect of the principle in Section 2. The linear relation between *E*_rms_ and E*_rms_ can be expressed as

(11) $$E_{\text{rms}}=k_{b}E_{\text{rms}}^{*}$$

where *k_b_* is the correction coefficient regarding the influence of the human body on measurement; it is the same as that in Equation (9). Here, *k_b_* = 1.8010.

Considering the dual influence of the measuring sensor and human body, we can correct the electric field intensity measured with the portable Type LP-2000 twice on the basis of Equations (10) and (11) to reflect accurately the original environmental electric field.

## 4. Experimental Verification

### 4.1. Experimental Result and Analysis in the Absence of a Human Body

**Figure 9:**
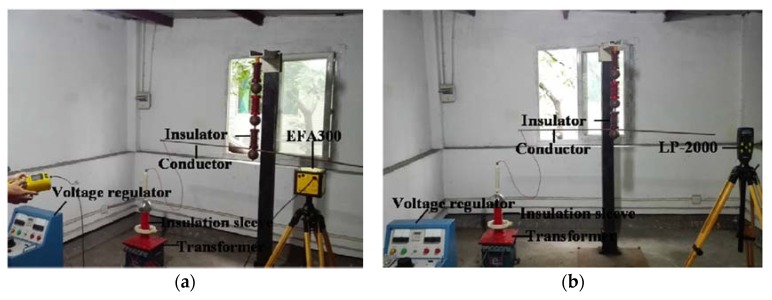
Experimental measurement (in the absence of a human body). (**a**) Measuring worksite of Narda EFA-300; (**b**) measuring worksite of Type LP-2000.

The experimental platform composed of a voltage regulator, step-up transformer, conductor and insulator is shown in Figure 9. Narda EFA-300 and a portable Type LP-2000 are used as the measurement devices. The technical characteristics of the equipment are shown in Table 4.

In the absence of a human body, the comparisons are conducted among the measured result *E*_LP-2000_ of Type LP-2000, the measured result *E*_EFA-300_ of Narda EFA-300, and the original electric field *E*_0_ obtained via stimulation. The measuring errors are defined as

(12a) $$e_{1}=\frac{\left|E_{\text{LP-2000}}-E_{0}\right|}{E_{0}}\times 100\%$$

(12b) $$e_{2}=\frac{\left|E_{\text{EFA-300}}-E_{0}\right|}{E_{0}}\times 100\%$$

The statistical data and error analyses are shown in Table 5.

**Figure 10:**
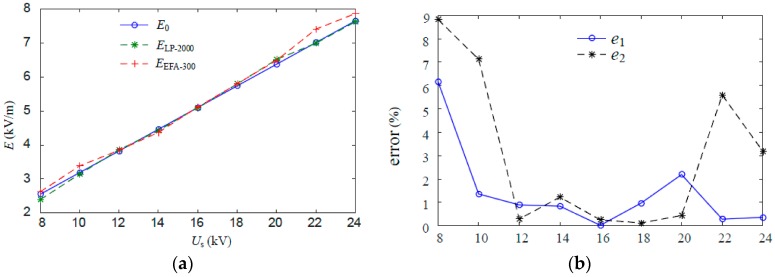
Relation curves related to the excitation voltage variation (in the absence of a human body). (**a**) Curves of electric field intensity; (**b**) curves of error.

Figure 10 shows the fitting curve based on the data in Table 5.

Given the interference produced by other electric equipment in the laboratory, certain errors emerge between the measured electric field intensity and the stimulation results. However, the value measured by Type LP-2000, in general, is relatively close to the stimulation value and the value measured by Narda EFA-300. Therefore, the accuracy of Type LP-2000 is verified.

### 4.2. Experimental Results and Analysis in the Case of an Approaching Human Body

#### 4.2.1. Regulating the Excitation Voltages of Electric Field

**Figure 11:**
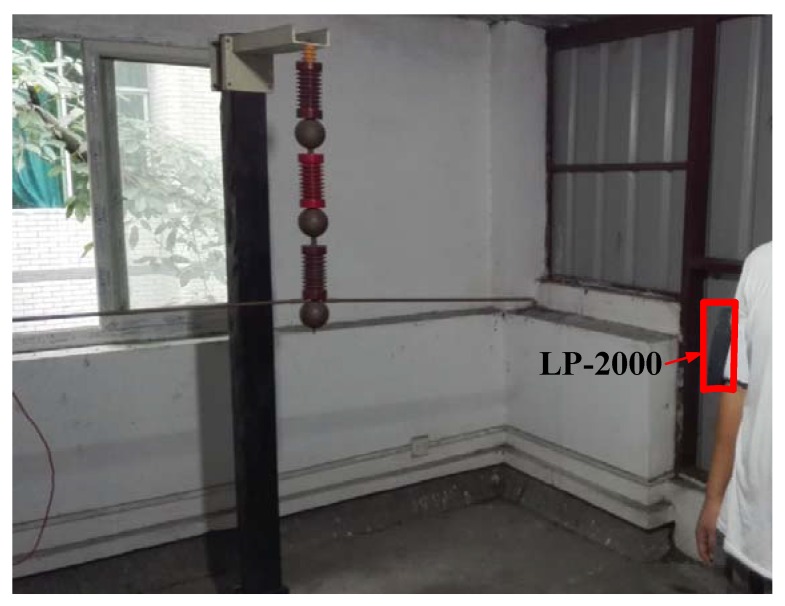
Measuring worksite of Type LP-2000 (in the case of an approaching human body).

Figure 11 shows that the Type LP-2000 sensor is fixed on the arm of the individual at a horizontal distance of 53 cm from the conductor. Different electric field intensities can be generated by regulating the excitation voltages of the conductor. The uncorrected measurement data E_LP-2000_ of Type LP-2000 and the original electric field E_0_ in the stimulation are listed in Table 6.

**Figure 12:**
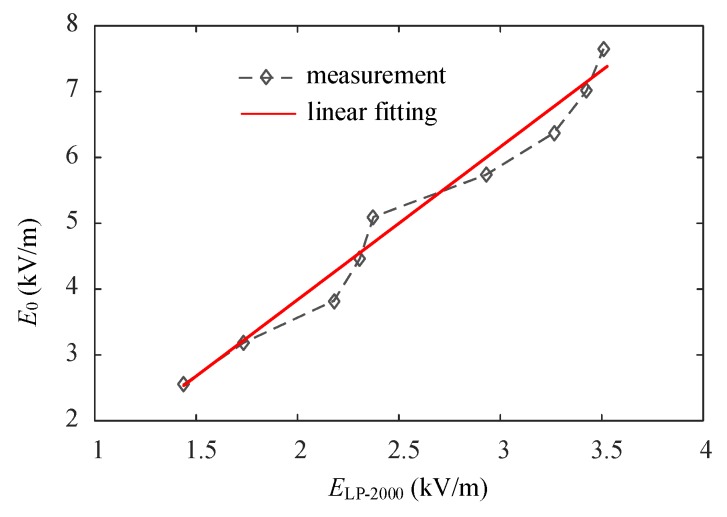
Relation curve of stimulation and uncorrected experimental data (in the case of an approaching human body).

Figure 12 shows the fitting curve based on the data in Table 6.

Figure 12 shows that the uncorrected measured electric field intensity in the case of an approaching human body is less than the original electric field intensity. Furthermore, an approximately linear relation exists between them. The linear coefficient can be obtained by linear fitting, *i.e*., *k*_b_* = 1.9094, which is close to *k*_b_ = 1.8010 as obtained in the stimulation. The error results from the electromagnetic interference in the experimental environment.

The measured result is corrected to obtain the corresponding E*_LP-2000_ by using the correction coefficient *k*_b_* = 1.9094. The comparison between the corrected measured data E*_LP-2000_ and the original electric field *E*_0_ is shown in Table 7.

Table 7 shows that the measured result of Type LP-2000 after correction accurately reflects the actual electric field intensity in the environment and that the maximal error is less than 6%.

#### 4.2.2. Regulating the Distances between the Measuring Point and the Conductor

**Figure 13:**
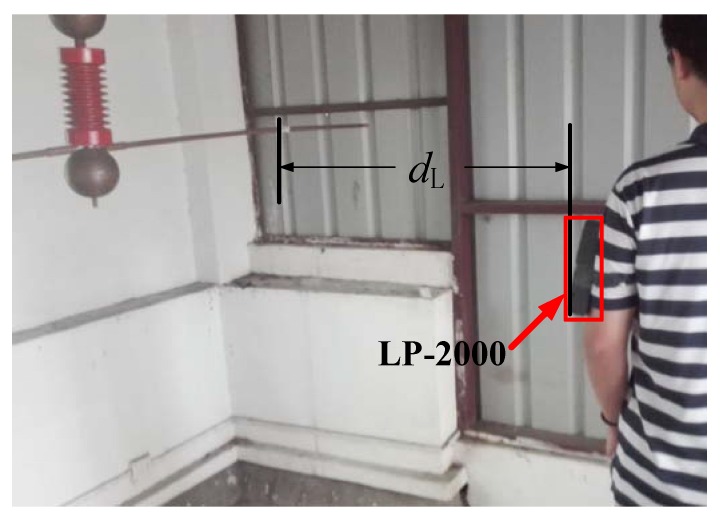
Electric field measurements in different distances (in the case of an approaching human body).

As most of the experimental conditions are kept identical to those in the previous experiment and the excitation voltage of the conductor is set to 12 kV, different electric field intensities can be obtained at different measuring points by regulating the distances between the measuring point and the conductor, as shown in Figure 13. The corrected measured data E*_LP-2000_ are obtained by using the correction coefficient *k*_b_* = 1.9094. The original electric field *E*_0_ in the simulation and the corresponding error values are listed in Table 8.

Table 8 also shows that the corrected electric field intensity measured by Type LP-2000 is consistent with the original electric field intensity in the environment and that the maximal error is less than 5%.

## 5. Conclusions

The influence of the human body on electric field measurement was investigated by using the Type LP-2000 single-shaft electric field measuring sensor developed by our research team. The results obtained from the principal model, simulation, and physical experiment showed that as an individual approached the sensor, the measured electric field intensity became less than the original environmental electric field intensity in the absence of a human body; however, both of them were proportional. The original environmental electric field intensity was obtained by using the electric field intensity measured with the portable Type LP-2000 in real time and multiplying its value by the defined correction coefficient. The correction coefficient k_b_ obtained in the simulation was 1.8010, and the correction coefficient *k*_b_* obtained in the experiment was 1.9094; the two values can be considered approximately equal. Two experimental programs were established; under these programs, the excitation voltages and the distance measuring points were regulated to produce different electric field intensities. Using *k*_b_* = 1.9094, the corrected measured electric field intensity accurately reflected the original environmental electric field intensity, and the maximal error was less than 6% in all the data comparisons. These results verify the effectiveness of our proposed correction method.

Additionally, the single-shaft sensor proposed in this study may be more suitable for the electric field generated by transmission lines than for that generated in substation. Therefore, the 3D measurement sensor will be explored in our future research.

## Figures and Tables

**Table 1:** Exposure limits regarding power-frequency electric fields developed by relevant institutions and international organizations.

Name	Publish Time	Frequency (Hz)	*E* (kV/m)	
			Occupational Exposure	Public Exposure
ICNIRP ^1^	2010	50	10	5
		60	8.3	4.2
IEEE ^2^	2002	50	20	5
NRPB ^3^	2004	50	10	5
		60	8.3	4.2
EU ^4^	2004	50	10	—
		60	8.3	
	1999	50	—	5
		60		4.2

1. ICNIRP——International Commission on Non-Ionizing Radiation Protection. 2. IEEE——Institute of Electrical and Electronics Engineers. 3. NRPB——National Resources Planning Board. 4. EU——European Union.

**Table 2:** Comparison of electric field values at point *P* before and after placing the measuring sensor (driven by different voltages).

*U*_s_ (kV)	8	10	12	14	16	18	20	22	24
*E*_0_ (kV/m)	2.5491	3.1865	3.8237	4.4610	5.0984	5.7357	6.3732	7.0103	7.6476
*E*_rms_ (kV/m)	0.7852	0.9816	1.1779	1.3742	1.5705	1.7668	1.9631	2.1594	2.3557

**Table 3:** Comparison of measured electric field values at point *P* before and after the approach of a human body (driven by different voltages).

*U*_s_ (kV)	8	10	12	14	16	18	20	22	24
*E*_rms_ (kV/m)	0.7852	0.9816	1.1779	1.3742	1.5705	1.7668	1.9631	2.1594	2.3557
E*_rms_ (kV/m)	0.4360	0.5450	0.6540	0.7630	0.8720	0.9810	1.0900	1.1990	1.3081

**Table 4:** Technical characteristics of the experimental equipment.

Name	Technical Characteristics
voltage regulator	input: 220 V with power frequency adjustable range: 0–250 V rated capacity: 10 kVA
transformer	rated capacity: 10 kVA ratio: 200
EFA-300	measurable frequency range: 5 Hz–32 kHz measurable electric field range: 0.1 V/m–200 kV/m
LP-2000	measurable frequency range: 5 Hz–1 kHz measurable electric field range: 20 V/m–200 kV/m

**Table 5:** Measurement data statistics of stimulation and experiment and error analysis (in the absence of a human body).

*U*_s_ (kV)	8	10	12	14	16	18	20	22	24
*E*_0_ (kV/m)	2.5491	3.1865	3.8237	4.4610	5.0984	5.7357	6.3732	7.0103	7.6476
*E*_LP-2000_ (kV/m)	2.3921	3.1431	3.8580	4.4237	5.0997	5.7912	6.5125	6.9905	7.6198
*E*_EFA-300_ (kV/m)	2.6234	3.3840	3.8467	4.3699	5.1124	5.7983	6.4842	7.4047	7.8630
*e*_1_ (%)	6.16	1.36	0.89	0.84	0.02	0.97	2.19	0.28	0.36
*e*_2_ (%)	8.82	7.12	0.29	1.22	0.25	0.12	0.44	5.59	3.19

**Table 6:** Uncorrected data statistics with different excitation voltages (in the case of an approaching human body).

*U*_s_ (kV)	8	10	12	14	16	18	20	22	24
*E*_0_ (kV/m)	2.5491	3.1865	3.8237	4.4610	5.0984	5.7357	6.3732	7.0103	7.6476
*E*_LP-2000_ (kV/m)	1.4361	1.7331	2.1793	2.3066	2.3745	2.9281	3.2647	3.4245	3.5080

**Table 7:** Corrected data statistics with different excitation voltages (in the case of an approaching human body).

*U*_s_ (kV)	8	10	12	14	16	18	20	22	24
*E*_0_ (kV/m)	2.5491	3.1865	3.8237	4.4610	5.0984	5.7357	6.3732	7.0103	7.6476
E*_LP-2000_ (kV/m)	2.4452	3.3538	3.7979	4.3544	4.8430	5.8499	6.3979	6.8109	7.2634
error (%)	4.08	5.25	0.67	2.39	5.01	1.99	0.34	2.84	5.02

**Table 8:** Corrected data statistics with different measurement distances (in the case of an approaching human body).

*d*_L_ (cm)	53	63	73	83	93	103
*E*_0_ (kV)	3.8237	3.2423	2.7417	2.372	2.0744	1.8264
E*_LP-2000_ (kV)	3.8582	3.3455	2.7956	2.3144	1.9733	1.7836
error (%)	0.8970	3.1675	1.9441	2.4452	4.8882	2.3763
